# Carotid Extra-Media Thickness in Children: Relationships With Cardiometabolic Risk Factors and Endothelial Function

**DOI:** 10.3389/fendo.2020.574216

**Published:** 2020-09-24

**Authors:** Lucia Pacifico, Francesco Massimo Perla, Luciana Tromba, Giovanni Carbotta, Michela Lavorato, Pasquale Pierimarchi, Claudio Chiesa

**Affiliations:** ^1^Department of Mother and Child Health, Sapienza University of Rome, Rome, Italy; ^2^Department of Surgical Sciences, Sapienza University of Rome, Rome, Italy; ^3^Institute of Translational Pharmacology, National Research Council, Rome, Italy

**Keywords:** carotid extra-media thickness, cardiometabolic risk factors, metabolic syndrome, NAFLD, endothelial function, youths

## Abstract

**Background:** Emerging evidence suggests that structural adventitial modifications and perivascular adipose tissue (PAT) may have a role in early atherogenesis. In a cohort of children and adolescents, we explored (1) the association of carotid extra-media thickness (cEMT), an ultrasound measure whose main determinants are arterial adventitia and PAT, with obesity and its cardiometabolic complications; and (2) the interplay between cEMT and endothelial function.

**Methods:** The study participants included 286 youths (age, 6–16 years; 154 boys, and 132 girls). Anthropometric and laboratory parameters, liver ultrasound, vascular structure measures [cEMT and carotid intima-media thickness (cIMT)], endothelial function [brachial artery flow-mediated dilation (FMD)] were obtained in all subjects. Non-alcoholic fatty liver disease (NAFLD) was diagnosed in the presence of hepatic fat on ultrasonography, in the absence of other causes of liver disease. Diagnosis of metabolic syndrome (MetS) was established on the basis of three or more of the following cardiovascular disease (CVD) risk variables: abdominal obesity, high triglycerides, low high-density lipoprotein cholesterol, elevated blood pressure (BP), and impaired fasting glucose.

**Results:** cEMT demonstrated significant associations with body-mass index (BMI) and waist circumference (WC), BP, insulin resistance, NAFLD, and inflammation. No association was found between cEMT and lipid values, and between cEMT and MetS. A stepwise multivariate linear regression analysis indicated that WC (β coefficient, 0.35; *P* < 0.0001) was the only determinant of cEMT, independently of other major cardiometabolic risk factors. Further adjustment for cIMT did not significantly alter this association. FMD was correlated to age, Tanner stage, total and abdominal obesity, BP, NAFLD, and cEMT. The association between FMD and cEMT was independent of age, sex, Tanner stage, WC, and BMI (β coefficient, −0.14; *P* = 0.027). After controlling for CVD risk factors and basal brachial artery diameter, cEMT remained associated with FMD (β coefficient, −0.11; *P* = 0.049).

**Conclusions:** In youths, cEMT is associated with abdominal fat, a well-established body fat depot with important implications for cardiovascular diseases. Furthermore, cEMT is related to FMD, suggesting that arterial adventitia and PAT may be involved in the early changes in endothelial function.

## Introduction

Childhood obesity represents one of the most important public health issues, tracking from childhood to adulthood and increasing the risk of cardiovascular disease (CVD) later in young adulthood (1). Because obesity is linked to comorbid conditions such as dyslipidemia, insulin resistance, hypertension, and inflammation since early life, these risk factors represent intermediate steps favoring the atherogenic process that starts in childhood, progresses throughout the lifespan and results in overt CVD (2, 3).

Previous non-invasive investigations on functional and anatomic vascular changes in children and adolescents have found endothelial dysfunction and adverse thickening of vascular wall as key indicators of potential CVD risk factors, consistent with their role in the early development of atherogenesis (4–6). Substantial evidence displays that the atherosclerotic disease process also involves structural abnormalities of the arterial adventitia following exposure to CVD risk factors, and that this event precedes structural alterations of the arterial intima and media in the early stages of atherosclerosis (7–10). Therefore, the assessment of arterial adventitia may add further insight into the vascular health related to CVD risk that is not predicted by evaluation of arterial intima media thickness (IMT) alone, possibly with distinctive associations of risk factors with arterial IMT and arterial adventitial thickness (11, 12). To this end, a new ultrasound parameter, carotid extra-media thickness (cEMT), was introduced especially as a marker of arterial adventitial thickness (11). The prominent components of cEMT are the arterial adventitia and perivascular adipose tissue (PAT) (13–15), being the most likely to be influenced by CVD risk factors (13, 16). Recent published studies have indicated that beside its structural role for blood vessels, PAT is also a physiologically and metabolically active endocrine tissue (17, 18). PAT has been found to secrete a number of adipokines and cytokines that influence endothelial function, vasoreactivity, and interfere with arterial wall structure. These findings raise the possibility that expansion of PAT in obesity could also be responsible of vascular disease at distant sites, in addition to its function as a local paracrine mediator.

Therefore, in a cohort of children and adolescents we aimed to (1) explore the association of cEMT with obesity and its complications including insulin resistance, metabolic syndrome (MetS), non-alcoholic fatty liver disease (NAFLD) and inflammation; and (2) investigate the interplay between cEMT and endothelial function.

## Methods

### Study Population

Over a 15-month period, 286 Caucasian youths were consecutively recruited through a screening program for CVD risk factors in childhood at the Mother and Child Health Department, Sapienza University of Rome. Participants with a history of chronic diseases (i.e., renal, endocrinologic, and hepatic disorders); diabetes mellitus; and conditions able to modify body composition as well as vascular function, were not included.

Physical examination including measurements of weight, standing height, body mass index (BMI), waist circumference (WC), assessment of the pubertal stage (19, 20), the grade of obesity (21), and systolic and diastolic blood pressure (BP), was performed in all enrollees as previously described (22, 23).

The Policlinico Umberto I Hospital Ethics Committee approved the study (protocol number 223/16), and the parents of all participants gave informed consent.

### Biochemical Data

Blood samples were obtained from each child after an overnight fast. Previously described methods (24) were used for the determination of circulating concentrations of glucose, insulin, triglycerides, total cholesterol, high-density lipoprotein cholesterol (HDL-C), low-density lipoprotein cholesterol (LDL-C), aspartate aminotransferase (AST), alanine aminotransferase (ALT), gamma-glutamyl transferase (GGT), and high-sensitivity C reactive protein (HSCRP).

### Carotid and Brachial Ultrasonography

Assessments of cEMT, carotid IMT (cIMT) and brachial artery flow-mediated dilation (FMD) were accomplished by an experienced vascular sonographer (LT), who was blinded to the clinical and biochemical data of the children. Measurement of cEMT was carried out employing high-resolution vascular ultrasonography (Mylab 70 XVision Gold, Esaote, Genova, Italy) equipped with 7.5–18-MHz linear-array transducer. Measures were acquired at the end-diastole from two cardiac cycles, and included the distance between the carotid media-adventitia border and the jugular wall-lumen interface (11, 13). The average of two measurements was used. The percentage variability between two repeated cEMT measurements on the same subjects [that were done in 32 randomly selected study children (17 obese, and 15 normal-weight)] was < 3.5%.

Assessment of cIMT was done as previously described (22, 23). Longitudinal ultrasound scans of the right and left common carotid arteries near the bifurcation at end-diastole were obtained on the same day as the studies of cEMT. The recorded cIMT value was an average of the cIMT results from the left and right sides. The day-to-day coefficient of variation from repeated scans for cIMT was found in this investigation as well as in previous studies from our research group to be <3% (22, 23, 25).

Ultrasound assessment of basal brachial artery diameter and endothelial-dependent FMD of brachial artery were investigated according to current guidelines (26), and as previously described (24, 27). Repeated FMD measurements in 15 randomly selected study children (10 overweight/obese, and 5 normal-weight) gave a very high intra-correlation coefficient (0.98).

### Hepatic Ultrasonography

Ultrasound examination of the liver was performed using a Toshiba Aplio XV scanner (Toshiba, Japan) equipped with 3.5–5-MHz convex transducers and tissue harmonics. Liver steatosis was defined as follows: a diffuse hyperechogenicity of the liver parenchyma compared to the renal cortex and spleen, deep beam attenuation, poor visualization of the diaphragm, and poor visualization of the intrahepatic architecture (23).

### Definitions

Overweight was defined as BMI ≥ 85th and < 95th percentile for age and sex, and obesity as BMI ≥ 95th percentile for age and sex. NAFLD was diagnosed in the presence of hepatic fat on ultrasonography, in the absence of other causes of liver disease. MetS was defined on the basis of three or more of the following CVD risk factors: abdominal obesity (28); high triglycerides (29); low HDL-C (29); elevated BP (30); impaired fasting glucose (glucose ≥ 5.6 mmol/l).

Insulin resistance was estimated by a homeostasis model assessment of insulin resistance (HOMA-IR). An elevated HOMA-IR value (≥ 90th percentile for age, sex, and BMI category) served as evidence of insulin resistance (31).

### Statistical Analysis

Results are presented as frequencies and as means with 95% confidence intervals (CI). Normality of data was assessed with the Kolmogorov-Smirnov test. cEMT, cIMT, insulin, HOMA-IR, and HSCRP values were non-normally distributed and log-transformed for analysis. Demographic, anthropometric and cardiometabolic differences across gender were compared by *t-*test for quantitative variables, and using X2 test for qualitative variables. Differences among normal-weight, overweight, and obese subjects were evaluated by one-way analysis of variance (ANOVA) for quantitative variables with the Bonferroni correction for multiple comparisons, and using X2 test for qualitative variables. The relationship between variables was assessed by Pearson's correlations and linear regression. The independence of the association of cEMT with cardiometabolic risk factors, after adjustment for age, gender and Tanner stage was determined by multiple linear regression analysis. Multiple linear regression analysis was also used to evaluate the association between FMD and cEMT, after adjustment for age, gender, Tanner stage and CVD risk factors. A *P* < 0.05 was considered statistically significant.

## Results

### Characteristics of the Study Population

Clinical, metabolic and cardiovascular characteristics of participants divided by sex are presented in Table 1. No gender-specific significant differences were observed for age, BMI, BMI-standard deviation score (SDS), WC, systolic and diastolic BP, lipid profile, GGT, fasting glucose and insulin, HOMA-IR values, and HSCRP. Males had higher AST and ALT levels than females. cEMT, cIMT, basal and peak brachial artery diameters, and FMD were slightly higher in boys than in girls, but the difference did not reach statistical significance.

**Table 1:** Characteristics of study population stratified by gender.

	**Boys** **(*n =* 154)**	**Girls** **(*n =* 132)**
**Anthropometric variables**		
Age, years	10.8 (10.3–11.2)	11.2 (10.6–11.6)
Prepubertal, n (%)	52 (33.8)	36 (27.3)
BMI	22.0 (21.0–22.9)	21.9 (21.0–22.8)
BMI-SDS	0.99 (0.79–1.18)	0.89 (0.70–1.07)
Waist circumference, cm	74.2 (71.7–76.7)	72.3 (69.9–74.6)
**Metabolic variables**		
Triglycerides, mg/dL	82 (75–88)	85 (78–92)
HDL-C, mg/dL	55 (53–57)	57 (55–60)
LDL-C, mg/dL	97 (91–103)	104 (98–110)
Aspartate aminotransferase, U/L	26 (24–27)	22 (21–24)^**^
Alanine aminotransferase, U/L	24 (20–27)	18 (15–21)^*^
γ-glutamyl transferase, U/L	14 (13–15)	13 (12–14)
Fasting glucose, mmol/L	84 (82–86)	82 (81–84)
Fasting insulin, μU/mL	15.7 (12.1–19.3)	14.7 (13.0–16.4)
HOMA-IR values	3.3 (2.5–4.1)	3.0 (2.6–3.4)
HSCRP, μg/L	898 (743–1,085)	765 (631–925)
**Cardiovascular variables**		
Systolic blood pressure, mmHg	105 (103–108)	104 (102–106)
Diastolic blood pressure, mmHg	66 (65–68)	65 (63–67)
cEMT, mm	0.61 (0.59–0.63)	0.59 (0.57–0.61)
cIMT, mm	0.55 (0.53–0.57)	0.53 (0.51–0.55)
Basal brachial artery diameter, mm	3.6 (3.5–3.7)	3.5 (3.4–3.6)
Peak brachial artery diameter, mm	4.0 (3.9–4.1)	3.9 (3.8–4.0)
FMD, %	12.7 (11.2–14.5)	11.9 (10.2–13.9)

*Results are expressed as n (%), mean (95% CI), or geometric mean (95% CI) for log-transformed variables*.

* *P < 0.05*;

** *P < 0.01*.

*BMI, body mass index; BMI-SDS, BMI-standard deviation score; cEMT, carotid extra-media thickness; CI, confidence intervals; cIMT, carotid intima-media thickness; FMD, flow-mediated dilation of brachial artery; HDL-C, high-density lipoprotein cholesterol; HOMA-IR, homeostasis model assessment of insulin resistance; HSCRP, high-sensitivity C reactive protein; LDL-C, low-density lipoprotein cholesterol*.

Based on BMI data, 36.7% (95% CI, 31.1–42.6) of participants were classified as obese, 17.8% (95% CI, 13.6–22.8) as overweight, and 45.5% (95% CI, 39.6–51.6) as normal-weight. The main anthropometric, metabolic and cardiovascular variables of the three study groups according to their BMI status (obese, overweight, and normal-weight) are summarized in Supplementary Table 1. As expected, cardiovascular and metabolic risk factors increased with increasing BMI.

Of the entire study population, 32.2 % (95% CI, 26.8–37.9) were centrally obese (Table 2). In Table 2, the proportions of children with NAFLD, MetS, and its individual components with the inclusion of insulin resistance are also shown.

**Table 2:** Prevalence of cardiometabolic risk factors in participants.

	**% (95% CI)**
**Obesity**	
BMI ≥ 95% percentile for age and gender	36.7 (31.1–42.6)
**Central obesity**	
Waist circumference ≥ 90th percentile for age and sex	32.2 (26.8–37.9)
**Dyslipidemia**	
HDL-C <40 mg/dL	7.7 (4.9–11.4)
Triglycerides ≥ 100 mg/dL in children, ≥ 130 mg/dL in adolescents	17.1 (13.0–22.0)
**Elevated blood pressure**	
Systolic and/or diastolic BP ≥ 90th percentile for age, sex, and height	28.3 (23.2–33.9)
**Impaired fasting glucose**	
Glucose ≥ 5.6 mmol/L	2.4 (1.0–5.0)
**Insulin resistance**	
HOMA-IR values ≥ 90th percentile for age, sex, and BMI category	27.3 (22.2–32.8)
NAFLD	18.9 (14.5–23.9)
Metabolic syndrome	21.7 (17.0–26.9)

*BMI, body mass index; BP, blood pressure; HDL-C, high-density lipoprotein cholesterol; HOMA-IR, homeostasis model assessment of insulin resistance; NAFLD, non-alcoholic fatty liver disease*.

### cEMT and Cardiovascular Risk Factors

**Figure 1:**
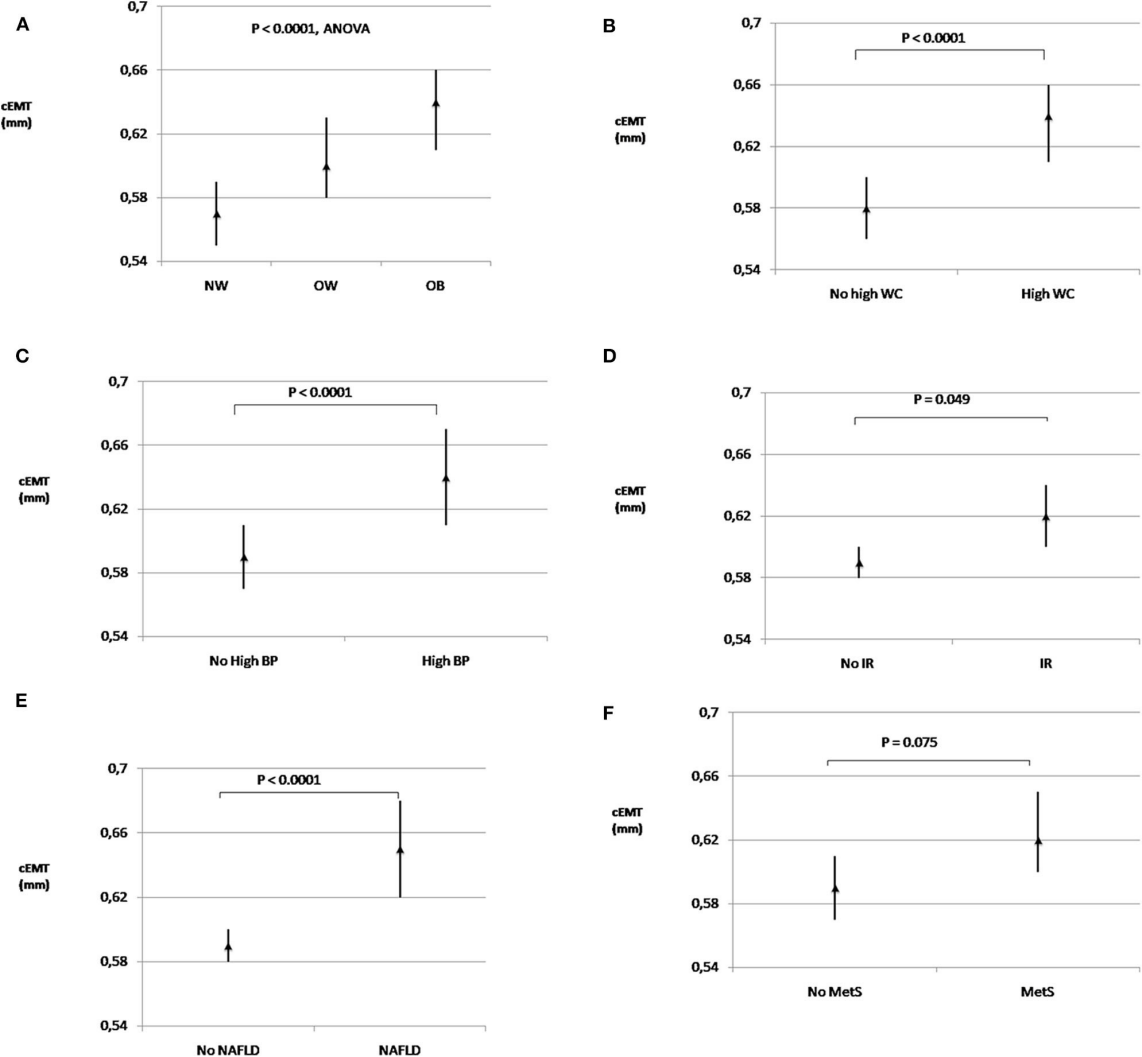
Extra-media thickness and cardiometabolic risk factors. Geometric mean values (95% confidence intervals) of carotid extra-media thickness in subjects with normal-weight, overweight and obesity **(A)**; with and without high WC **(B)**; with and without high BP **(C)**; with and without IR **(D)**; with and without NAFLD **(E)**, and with and without MetS **(F)**. cEMT, carotid extra-media thickness; BP, blood pressure; IR, insulin resistance; MetS, metabolic syndrome; NAFLD, non-alcoholic fatty liver disease; NW, normal-weight; OB, obesity; OW, overweight; WC, waist circumference.

Significant differences in cEMT emerged among normal-weight, overweight, and obese youths [0.57 (95% CI, 0.55–0.59) vs. 0.60 (0.58–0.63) vs. 0.64 (0.61–0.66); *P* < 0.0001, respectively] (Figure 1A). cEMT was significantly greater in youths with central obesity than in those without [0.64 (0.61–0.66) vs. 0.58 (0.57–0.60); *P* < 0.0001] (Figure 1B); in children with elevated BP compared to those with normal BP [0.64 (0.61–0.67) vs. 0.59 (0.58–0.60); *P* < 0.0001] (Figure 1C); in subjects with insulin resistance than in those without [0.62 (0.60–0.64) vs. 0.59 (0.58–0.61); *P* < 0.05] (Figure 1D); and in patients with NAFLD compared to those without liver involvement [0.66 (0.62–0.68) vs. 0.59 (0.58–0.60); *P* < 0.0001] (Figure 1E). In contrast, no significant differences in geometric mean values of cEMT were observed in children with high triglycerides and low HDL-C concentrations in comparison with those without lipid alterations. cEMT values were higher in subjects with MetS [0.62 (0.60–0.65)] than in subjects without MetS [0.59 (0.57–0.61)], displaying a near-significant association (*P* = 0.075) (Figure 1F).

The associations of cEMT with the aforementioned CVD risk factors were also confirmed after controlling for the effects of age, sex, and Tanner stage (Table 3). In fact, cEMT demonstrated significant associations with BMI, BMI-SDS, WC, systolic and diastolic BP, insulin resistance, NAFLD, and HSCRP. A stepwise multivariate linear regression analysis, in which all the significant variables were included, indicated that WC [B (95% CI), 0.004 (0.003–0.006); β coefficient, 0.35; *P* < 0.0001] was the only independent determinant of cEMT. Further adjustment for cIMT in the multivariate model did not significantly alter this association [B (95% CI), 0.002 (0.001–0.004); β coefficient, 0.21; *P* < 0.0001].

**Table 3:** Age-, sex-, and tanner –adjusted linear regression analyses for associations^#^ between cardiovascular risk factors and carotid measures.

		cEMT^*^		
	**B**	**Beta coefficient**	***P*-value**	**95% CI**
**BMI, kg/m**^**2**^	**0.009**	**0.28**	**<** **0.0001**	**0.005, 0.013**
**BMI-SDS**	**0.04**	**0.27**	**<** **0.0001**	**0.023, 0.057**
**WC, cm**	**0.004**	**0.30**	**<** **0.0001**	**0.002, 0.005**
**Systolic BP, mmHg**	**0.003**	**0.27**	**<** **0.0001**	**0.002, 0.005**
**Diastolic BP, mmHg**	**0.003**	**0.17**	**0.014**	**0.001, 0.005**
HDL-C, mg/dL	−0.001	−0.11	0.082	−0.003, 0.000
LDL-C, mg/dL	0.000	0.036	0.55	−0.001, 0.000
Triglycerides, mg/dL	0.000	0.008	0.89	0.000, 0.000
Glucose, mmol/L	0.001	0.054	0.37	−0.001, 0.003
**HOMA-IR values**	**0.045**	**0.20**	**0.002**	**0.017, 0.074**
**HSCRP**, **μg/L**	**0.028**	**0.19**	**0.002**	**0.011, 0.045**
**NAFLD**	**0.08**	**0.18**	**0.003**	**0.028, 0.133**
Metabolic syndrome	0.03	0.071	0.24	−0.021, 0.08

# *Significant associations are highlighted in bold*.

* *log values*.

*BMI, body mass index; BMI-SDS, body mass index-standard deviation score; BP, blood pressure; cEMT, carotid extra-media thickness; cIMT, carotid intima media thickness; HDL-C, high-density lipoprotein cholesterol; HOMA-IR, homeostasis model assessment of insulin resistance; HSCRP, high-sensitivity C reactive protein; LDL-C, low-density lipoprotein cholesterol; NAFLD, non-alcoholic fatty liver disease; WC, waist circumference*.

### cEMT and Endothelial Function

In univariate analysis FMD was correlated to age (*r* = –0.15; *P* = 0.017), Tanner stage (*r* = –0.14; *P* = 0.021), BMI (*r* = –0.14; *P* = 0.024), WC (*r* = –0.16; *P* = 0.01), systolic BP (*r* = –0.15; *P* = 0.015), diastolic BP (*r* = –0.20; *P* = 0.001), NAFLD (*r* = –0.16; *P* = 0.01), and cEMT (*r* = –0.17; *P* = 0.008). The association between FMD and cEMT was independent of age, sex, and Tanner stage (β coefficient, –0.15; *P* = 0.014). Further adjustment for WC and BMI did not markedly change the effect of this association (β coefficient, –0.14; *P* = 0.027). In a stepwise multivariate linear regression analysis, in which all the CVD risk factors and basal brachial artery diameter were included, cEMT remained associated with FMD (β coefficient, –0.11; *P* = 0.049).

## Discussion

Findings of this cross-sectional study in youths showed that cEMT correlates with several CVD risk factors including general and abdominal obesity, elevated BP, insulin resistance, and NAFLD, independently of the effects of age, sex, and Tanner stage. In multiple regression, only abdominal obesity remained statistically significantly associated with cEMT after adjustment for other cardiometabolic risk factors. Thus, our findings do point to abdominal fat accumulation, a well-established body fat depot with important implications for cardiovascular diseases, as independent predictor of cEMT in childhood.

Substantial evidence indicates that cEMT incorporates not only the arterial adventitial thickness, but also PAT, interstitial tissue, and the whole wall of jugular vein. With the exception of PAT, these non-adventitial components are unlikely to be altered by CVD risk factors (13, 32). Though earlier studies by Skilton et al. (11) have proposed that EMT is an index of arterial adventitia, Falk et al. (15) demonstrated that the non-adventitial components are an important contributor to cEMT, and that cEMT consists of a considerable quota of adipose tissue within or around adventitia, in particular in obese subjects. Thus, considering that perivascular fat deposition is a correlate of visceral adiposity (33, 34), it is not surprising that we found a significant association between abdominal obesity and cEMT. Accordingly, Haberka et al. (14) evaluated prospectively cEMT in 400 patients with a high CVD risk admitted to the Department of Cardiology for planned cardiovascular diagnostics. They found that cEMT was associated with several parameters of general obesity and abdominal fat distribution driven by abdominal visceral fat. In this regard, cEMT correlated with WC with better coefficients when ultrasound-derived intraabdominal thickness was used compared to ultrasound-measured preperitoneal fat layer. Yet, Lefferts et al. (35) who retrospectively assessed cEMT (from carotid pictures acquired expressly for cIMT measurement) in a group of 135 young healthy males (of whom 55% were normal-weight and 45% overweight or obese), reported significant associations between markers of visceral adiposity (such as WC and sagittal abdominal diameter) and cEMT.

Because atherosclerosis frequently starts at a very young age and persists over time (36), studies undertaken in early life are important. However, there are yet no studies on gender- and age-specific physiological values of cEMT in childhood that may facilitate correct interpretation of data in both research and clinical settings. Moreover, scant information is available on the association between cEMT and CVD risk factors in childhood. The first study involving 389 8-year-old youths without diabetes, demonstrated an association between cEMT and gender, height, adiposity, BP, and family history of premature CVD (12). Beside a gender-specific relationship of weight with cEMT among boys, the associations of gender, BP, and height with cEMT were independent of cIMT. The second study involving 379 8-year-old children without diabetes who had full data available for birth weight, gestational age, early post-natal weight gain and cEMT, found that increased cEMT was related to excessive weight gain over infancy (13). The limitation of these two studies was that cEMT was analyzed on images obtained specifically for the assessment of cIMT. In the third study including a small group of children and adolescents (38 obese, and 30 age-, and sex-matched healthy controls), it was found that cEMT was higher in adolescents vs. children and in obese subjects vs. non-obese controls, but it did not differ according to sex (37). In the same study, metabolic parameters related to obesity were also determined, but BMI remained the only independent risk factor affecting cEMT even after adjusting for cIMT. Thus, our finding of an independent association between cEMT and visceral fat has not been documented previously in the pediatric population.

To our knowledge, our study is the first to analyze in youths the relationship between extra-media changes and endothelial function. cEMT was associated with FMD, suggesting that arterial adventitia and PAT (encapsulated by cEMT) may be involved in early changes in endothelial function. It is well-known that the endothelium plays a central role in the process of atherosclerosis. In this regard, endothelial dysfunction, patterned by deregulation of vasoreactivity, increased inflammation and oxidative stress, as well as by impaired barrier function, has been reported to precede the development of atherosclerotic disease, and to be associated with CVD (38–40). Interestingly, growing evidence suggests that PAT and adventitial inflammation pave the way for endothelial dysfunction and atherosclerotic plaque formation (41). It is now well-accepted that PAT does not simply reflect changes in visceral adipose tissue as seen in obesity, but has unique characteristics with profound effects on vascular biology (42). Under physiologic conditions PAT exerts anti-inflammatory (through secretion of protective adipokines) and vasodilatory (through a nitric oxide-dependent mechanism and a direct action on small muscle cells) functions (42–44). In contrast, PAT becomes dysfunctional under pathologic conditions (obesity, diabetes, hyperlipidemia) reducing expression of vasorelaxing substances and increasing vasoconstricting agents with infiltration of inflammatory cells leading to inflammatory state and oxidative stress (42–45). Thus, inflammation and oxidative stress, which are interconnected with adipose tissue dysfunction, contribute to endothelial dysfunction as seen in many pathologic conditions including obesity (43, 44, 46). Further research is needed to clarify the interplay between cEMT and endothelial function.

The current study has some strengths: (a) it includes a relatively large cohort of children and adolescents, and a complete analysis of metabolic variables; (b) it presents the first results concerning both cEMT and endothelial dysfunction in a cohort of youths. On the other hand, some limitations should also be taken into account: (a) the cross-sectional nature of the study, that may not provide definite information about cause-effect relationship. Only longitudinal studies will give more information and confirmation of the predictive value of cEMT to estimate cardiometabolic risk; (b) the tissue region of interest, where cEMT was measured by ultrasound, includes not only PAT and we cannot distinguish the relative contribution of PAT and adventitia, respectively, on cardiovascular health in our study population; and (c) failure to measure key adipokines, cytokines, and inflammatory markers that would have strengthened study results.

In conclusion, our data in children and adolescents indicate that cEMT (a) is correlated with visceral adipose tissue and thus with systemic metabolic changes associated with obesity; and (b) is an independent predictor of changes in endothelial function. Finally, our study does not indicate that cEMT is a more important or better measure of vascular structure than cIMT; instead, our data indicate that cEMT and cIMT represent different pathophysiological processes that can be assessed independently and might provide additional indication of how the structural modification of the vasculature impacts function. Considering the worldwide increase of pediatric obesity, measurements of cEMT may be useful to detect among obese children those with high CVD risk who may require thorough medical examination, and intensive lifestyle changes for prevention of atherosclerosis progression.

## Data Availability Statement

The raw data supporting the conclusions of this article will be made available by the authors, without undue reservation.

## Ethics Statement

The studies involving human participants were reviewed and approved by The Policlinico Umberto I Hospital Ethics Committee. Written informed consent to participate in this study was provided by the participants' legal guardian/next of kin.

## Author Contributions

LP, FP, LT, PP, and CC conceptualized and designed the study. LP, FP, LT, GC, and ML collected the data. LP, ML, PP, and CC analyzed and interpreted the data. LP and CC wrote the manuscript. All authors participated to the discussion of results and critically commented the manuscript for the final approval.

## Conflict of Interest

The authors declare that the research was conducted in the absence of any commercial or financial relationships that could be construed as a potential conflict of interest.
