# Supplementary material for: Carotid Extra-Media Thickness in Children: Relationships With Cardiometabolic Risk Factors and Endothelial Function
Source: Front Endocrinol (Lausanne). 2020 Sep 24;11:574216. doi: 10.3389/fendo.2020.574216 (PMC7541844; doi:10.3389/fendo.2020.574216)
Supplement: Supplementary file 1 [file Table_1.docx]

**Supplementary Table 1. Characteristics of study children stratified by BMI status**

|  | Normal-weight  (n= 130) | Overweight  (n= 51) | Obese  (n= 105) | P value |
| --- | --- | --- | --- | --- |
| **Anthropometric variables** |  |  |  |  |
| Age, years | 10.7 (10.2-11.3) | 11.0 (10.4-11.7) | 11.2 (10.5-11.7) | 0.55 |
| Prepubertal, n (%) | 48 (36.9) | 12 (23.5) | 28 (26.6) | 0.29 |
| BMI | 17.5 (17.0-17.9) | 22.8 (21.1-23.6) # | 27.7 (26.8-28.5) #,$ | < 0.0001 |
| BMI-SDS | -0.10 (-0.22-0.26) | 1.48 (1.37-1.58) # | 2.10 (2.04-2.16) #,$ | < 0.0001 |
| Waist circumference, cm | 62.0 (60.5-63.5) | 76.6 (74.5-78.8) # | 87.3 (85.0-89.5) #,$ | < 0.0001 |
| **Metabolic variables** |  |  |  |  |
| Triglycerides, mg/dL | 66 (62-71) | 93 (81-105) & | 101 (91-111) # | < 0.0001 |
| HDL-C, mg/dL | 62 (60-64) | 53 (50-56) # | 50 (48-53) # | < 0.0001 |
| LDL-C, mg/dL | 94 (87-99) | 104 (96-111) | 107 (97-117)***** | 0.02 |
| Aspartate aminotransferase, U/L | 16 (21-24) | 23 (22-26) | 25(22-27) | 0.88 |
| Alanine aminotransferase, U/L | 16 (15-17) | 22 (16-28) | 28 (22-34) # | < 0.0001 |
| γ-glutamyl transferase, U/L | 11 (10-12) | 15 (13-17) # | 15 (14-16) # | < 0.0001 |
| Fasting glucose, mmol/L | 83 (82-85) | 83 (82-85) | 82 (81-86) | 0.56 |
| Fasting insulin, µU/mL | 7.9 (7.2-8.6) | 11.8 (10.5-13.2) # | 19.9 (17.5-22.6) #,$ | < 0.0001 |
| HOMA-IR values | 1.6 (1.5-1.8) | 2.3 (1.9-2.7) & | 4.0 (3.4-4.6) #,$ | < 0.0001 |
| HSCRP, µg/L | 478 (399-567) | 1085 (812-1450) # | 1556 (1274-1900) # | < 0.0001 |
| **Cardiovascular variables** |  |  |  |  |
| Systolic blood pressure, mmHg | 98 (96-100) | 106 (103-109) # | 114 (111-117) #,$ | < 0.0001 |
| Diastolic blood pressure, mmHg | 61 (59-62) | 68 (66-71) # | 72 (69-74) # | < 0.0001 |
| cEMT, mm | 0.57 (0.55-0.59) | 0.60 (0.58-0.63)* | 0.64 (0.61-0.66) # | < 0.0001 |
| cIMT, mm | 0.53 (0.51-0.55) | 0.54 (0.52-0.56) | 0.57 (0.54-0.59)* | 0.019 |
| Basal brachial artery diameter, mm | 3.7 (3.5-3.8) | 3.5 (3.4-3.7) | 3.4 (3.3-3.7)+ | 0.004 |
| Peak brachial artery diameter, mm | 4.2 (4.0-4.3) | 4.0 (3.9-4.2) | 3.8. (3.7-3.9) # | < 0.0001 |
| FMD, % | 13.0 (11.3-15.0) | 11.4 (9.2-14.0) | 10.0 (8.4-12.3)* | 0.039 |

Results are expressed as n (%), mean (95% CI), or geometric mean (95% CI) for log-transformed variables.

P value by ANOVA with Bonferroni’s correction for multiple comparisons

*P<0.05; + P< 0.01; & P< 0.001; # P< 0.0001 vs normal-weight

$ P< 0.0001 vs overweight

BMI, body mass index; BMI-SDS, BMI-standard deviation score; cEMT, carotid extra-media thickness; CI, confidence intervals; cIMT, carotid intima-media thickness; FMD, flow-mediated dilation of brachial artery; HDL-C, high-density lipoprotein cholesterol; HOMA-IR, homeostasis model assessment of insulin resistance; HSCRP, high-sensitivity C reactive protein; LDL-C, low-density lipoprotein cholesterol.
